# Calpain inhibition as a novel therapeutic strategy for aortic dissection with acute lower extremity ischemia

**DOI:** 10.1186/s10020-025-01212-7

**Published:** 2025-04-21

**Authors:** Qiwen Tan, Xiaokang Wang, Wanchuang Xu, Kun Song, Yifan Xiong, Zhentong Jiang, Jingjing Li, Yunsheng Yu, Wenxue Ye, Zhenya Shen, Xiaomei Teng

**Affiliations:** 1 Department of Cardiovascular Surgery, First Affiliated Hospital of Soochow University, 899 Pinghai Road, Suzhou, 215006 China; 2 Suzhou Medical College, Soochow University, 199 Renai Road, Suzhou, 215123 China; 3 Institute for Cardiovascular Science, Soochow University, 178 Ganjiang Road, Suzhou, 215006 China

**Keywords:** Aortic dissection, Lower limb ischemia, Calpain, Fabp3

## Abstract

**Background:**

Aortic dissection (AD) patients with malperfusion present significant challenges and are associated with high postoperative mortality rates. Limited data exist regarding the management of patients with AD and acute lower extremity ischemia. Early diagnosis of the extent of malperfusion and timely intervention are critical for improving patient prognosis.

**Methods:**

A total of 104 patients diagnosed with AD were enrolled in this observational retrospective study, of which 11 (10.6%) presented with lower limb ischemia (LLI). A comparative analysis was conducted on the clinical data of the AD group and the AD + LLI group. Plasma concentrations of SBDP145, a specific indicator of Calpain activity, were quantified in Control, AD, and AD + LLI groups using ELISA. To explore the role of Calpain in LLI and AD, pharmacological inhibition with Calpeptin and transgenic mice overexpressing calpastatin (Tg-CAST) were utilized in mouse models. RNA sequencing and functional assays were employed to identify the downstream effectors of Calpain.

**Results:**

Patients in the AD + LLI group exhibited significantly elevated leukocyte counts, percentages of neutrophils and lymphocytes, as well as increased serum levels of AST, creatinine, total cholesterol, low-density lipoprotein, uric acid, and creatine kinase compared to those in the AD group. Furthermore, the mean calcium ion concentration and Ca^2+^-dependent Calpain activation were significantly higher in the AD + LLI patients. Both endogenous and exogenous Calpain inhibitors effectively promoted the restoration of blood flow to ischemic hind limbs by inhibiting the inflammatory response and promoting vascular regeneration. Additionally, Calpain inhibition prevented the onset and progression of AD. RNA sequencing and Western Blot assays demonstrated that Calpain inhibition significantly increased levels of Fabp3, which is involved in the ischemia-induced fatty acid metabolism pathway.

**Conclusions:**

Inhibition of Calpain has been demonstrated to decrease the incidence of AD and enhance the restoration of blood flow perfusion in ischemic lower extremities. This effect may be mediated by the upregulation of Fabp3. These findings highlight the potential for targeted interventions against Calpain as a novel therapeutic strategy in the treatment of cardiovascular disease.

**Supplementary Information:**

The online version contains supplementary material available at 10.1186/s10020-025-01212-7.

## Introduction

Aortic dissection (AD) is a serious cardiovascular condition characterized by a high mortality rate, which occurs when blood leaks through the lining of the aortic wall into the media, forming a false lumen. Untreated patients experience an alarming increase in mortality, escalating by 1–2% per hour, while the mortality rate for inpatients receiving clinical treatment remains significantly elevated at 26% (Minino et al. 2011). Globally, the incidence of AD ranges from 2 to 4 cases per 100,000 people per year, with males outnumbering females, and this incidence is on the rise (Lim and Khanafer 2023; Smedberg et al. 2024).

In China, the incidence of AD is estimated to be approximately 2.78 cases per 100,000, with a notably higher incidence in males compared to females (Tang et al. 2021). AD and its postoperative consequences can obstruct the aortic branch vessels, leading to inadequate perfusion of vital organs such as the brain, spinal cord, kidneys, mesentery, and limbs (Kamei and Kuriyama 2022; Lauterbach et al. 2001; Liu et al. 2002, 2024; Sickels et al. 2024; Wang et al. 2024). This condition is recognized as the second most common fatal complication of AD, following rupture (Norton et al. 2019). Specifically, lower limb ischemia (LLI) occurs in up to 8–10% of patients with acute aortic dissection, potentially resulting in permanent limb dysfunction or amputation if diagnosis and treatment are delayed (Norton et al. 2020). Consequently, early diagnosis of the extent of malperfusion and timely intervention are critical for improving patient prognosis (Goyal et al. 2024).

Calpain is a highly conserved family of calcium-dependent cysteine proteases that are ubiquitously expressed in all cell types. These proteases play crucial roles in various cellular processes, including the remodeling of cytoskeletal structures, membrane attachments, diverse signaling transduction pathways, and apoptosis (Goll et al. 2003). Numerous studies have demonstrated that the activation of the calpain system significantly contributes to the development of cardiovascular diseases, such as aortic aneurysm, atherosclerosis, and cardiomyopathy (Chattopadhyay et al. 2023; Muniappan et al. 2021; Potz et al. 2016). Calpain is implicated in endothelial cell dysfunction and promotes inflammatory immune responses (Cheetham et al. 2024; Miyazaki et al. 2020; Yan et al. 2024). However, the specific role of calpain in patients with aortic dissection, particularly regarding LLI, remains unclear.

In this study, we investigated the effects of Calpain inhibition on AD and acute lower extremity ischemia by analyzing clinical data from patients with AD, both with and without LLI. Furthermore, we examined the effects of Calpain inhibition in a mouse model of lower limb ischemia. The findings underscore the potential application of targeted interventions against Calpain as an emerging therapeutic strategy for AD and poor perfusion syndrome.

## Materials and methods

### Study population

This observational retrospective single-center study aimed to evaluate serum Calpain activity and clinical outcomes in patients with AD who presented with LLI. From March 2022 to March 2023, consecutive patients diagnosed with AD and treated at The First Affiliated Hospital of Soochow University were identified from the hospital’s database. Patients with AD and concomitant LLI were selected through retrospective analysis. Aortic dissection is primarily diagnosed by computed tomography angiography (CTA) images. The diagnosis of AD with concomitant LLI was based on clinical presentation, physical examination and radiographic findings. LLI was defined as the absence of femoral artery pulses, accompanied by one or more of the following symptoms: leg pain, pallor, paresthesia, or paralysis in the affected leg (Meisenbacher et al. 2022). Patients with penetrating aortic ulcers, aortic intramural hematomas, postoperative recurrences, and medical or traumatic aortic coarctation were excluded from the study. Additionally, patients with diabetic foot causing LLI, or peripheral vascular disease, such as atherosclerotic occlusive disease, thromboembolic vasculitis, and polyarteritis nodosa, were also excluded.

All patients underwent blood sample collection and physical examination within 24 h of admission. Baseline patient characteristics, blood indices, and postoperative outcome data were extracted from the institutional database and further supplemented by a retrospective review of medical records. The study was approved by the Ethics Committee of the First Affiliated Hospital of Soochow University (approval number: 2022 − 689). All clinical information and blood samples were obtained from the patients themselves with informed consent.

### ELISA

SBDP145 is a specific substrate of αII-spectrin, generated by Calpain-mediated cleavage, and serves as a reliable indicator of Calpain activity (Papa et al. 2018). The concentration of human serum SBDP145 was measured using a human αII-spectrin breakdown product SBDP145 ELISA kit (CSB-Eq. 028022HU, Cusabio). The ELISA procedure was performed in accordance with the manufacturer’s protocols.

### Animal experiments

Experiments were conducted using 6–8 weeks old male C57BL/6JGpt mice, which were purchased from GemPharmatech Co., Ltd. in Jiangsu, China. Additionally, transgenic mice (6–8 weeks old males) overexpressing calpastatin (TgCAST) were generously provided by Dr. TQ Peng (Li et al. 2018). All mice were housed under an alternating 12-hour light-dark cycle at a stable temperature of 22 ± 1 ℃ and were maintained on a standard chow diet with free access to food and water. All animal experiments adhered to the Guide for the Care and Use of Laboratory Animals published by the Institute for Laboratory Animal Research, National Research Council, Washington, DC (National Academy Press). All procedures were approved by the Soochow University Animal Research Ethics Committee.

### A mouse model of lower limb ischemia

Mice were anesthetized using isoflurane and ether inhalation. A surgical incision was made in the left inguinal region to expose the left femoral artery. The artery was isolated, and the fine arterial branches were disrupted, while the accompanying femoral nerve was separated. The distal and proximal branches of the femoral artery were ligated with 5 − 0 sterile sutures, and the segment of the femoral artery between the two ligation points was excised. The skin was then sutured after ensuring thorough hemostasis. The animal experiments were divided into two parts: the administration of the pharmacologic inhibitor Calpeptin and the overexpression of Calpastatin in transgenic mice (TgCAST).

The mice were divided into four groups: the Sham group, which underwent only skin incision and suturing of the left lower limb; the Sham + Cal group, which received an intraperitoneal injection of Calpeptin (0.02 mg/mL per g of body weight, administered once prior to surgery and three times within one week post-surgery (Tabata et al. 2010); the LLI group, which underwent lower limb ischemia surgery; and the LLI + Cal group, which consisted of mice that underwent lower limb ischemia surgery and received inhibitor injections at the same dosage and method.

Both wild-type (WT) and TgCAST mice underwent surgical procedures. Immediately following surgery, lower limb perfusion was assessed using a laser Doppler perfusion imager (MoorLDI-HIR). A perfusion ratio of the surgical side to the contralateral side of less than 20% was established as the criterion for successful induction of LLI (Yuan et al. 2023). Relative perfusion is defined as the ratio of the signal from the lower limb on the surgical side to that from the contralateral lower limb. Necrosis is considered to have occurred if the lower limb on the ischemic side of the mouse exhibits any of the following signs: blackening and loss of paw function, absence of toes, or an enlarged limb. The experimental process was illustrated in Fig. S1.

### A mouse model of aortic aneurysm or dissection

Three-week-old C57BL6/J mice were administered beta-aminopropionitrile (BAPN) at a dosage of 1 g/kg/day (Sigma-Aldrich, A3134) in their drinking water for four weeks, as described previously (Yang et al. 2023). Mice were randomly assigned to receive either a vehicle or Calpeptin (0.02 mg/mL per g of body weight) as described previously. Echocardiography was performed three weeks after the initiation of BAPN administration. AD was defined by the formation of a false lumen containing blood within the medial layer, while aortic aneurysm was classified as arterial dilation exceeding 50% of the normal diameter.

At the end of the experiments, all mice were euthanized under deep anesthesia with 100% O2/5% isoflurane, until respiratory arrest persisted for more than 60 s.

### Quantitative real-time PCR

Total RNA was extracted from the gastrocnemius muscle of mice using TRIzol Reagent (Invitrogen, CA, USA). cDNA synthesis was performed with the Primescript™RT Master Mix (TaKaRa, Japan), followed by quantitative PCR using the TB Green^®^ Premix Ex Taq™ II kit (TaKaRa, Japan). The primer sequences for all genes detected by qPCR were provided in Table S1.

### Western blot analysis

The gastrocnemius muscle was harvested from the ischemic limbs of mice and subsequently stored at -80 ℃. Western blot analysis was performed as follows: muscle tissue was lysed in RIPA buffer (Beyotime, China) with PMSF (Beyotime, ST506-2, China), and homogenized using a high-speed, low-temperature tissue homogenizer (Servicebio, China). Proteins (20 µg per sample) were separated by SDS-PAGE (Beyotime, China) and transferred to a PVDF membrane. The membrane was blocked with 5% whole milk for 1 h and then incubated with primary antibodies for 12 to 16 h at 4 ℃.

The antibodies included GAPDH (Proteintech, 1:10000, rabbit source), Calpain1 (Proteintech, 1:1000, rabbit source), Calpain2 (Proteintech, 1:1000, rabbit source), and Fabp3 (Proteintech, 1:5000, mouse source). After the primary antibody incubation, the membranes were treated with secondary antibodies (Proteintech, 1:10000, goat anti-rabbit or goat anti-mouse) for 1 h. Visualization of the blots was performed using the Odyssey imaging system (LI-COR Biosciences, USA) and quantification was carried out with ImageJ software.

### Histological and immunofluorescence staining

The gastrocnemius muscles were harvested and fixed in 4% paraformaldehyde for 24 h. The tissue samples were then embedded in paraffin and sectioned into slices with a thickness of 5 μm. Each slice underwent hematoxylin and eosin (H&E) staining, as well as Masson staining, to facilitate morphological analysis and fibrosis assessment. Quantitative analysis was performed using ImageJ to evaluate both the muscle bundle area and the collagen fiber area.

Tissue paraffin sections were deparaffinized, washed and subjected to antigen retrieval. The sections were then blocked with 5% goat serum and incubated with a primary antibody solution at 4 °C overnight. The primary antibodies included CD31 (zuochengbio, 1:100, rabbit source), α-SMA (zuochengbio, 1:50, mouse source), and Ki67 (zuochengbio, 1:400, rabbit source). After the incubation, Alexa Fluor^®^488 and Alexa Fluor^®^594 conjugated fluorescent secondary antibodies were applied. The sections were then stained with DAPI for 5 min. Images were captured with a Leica DMI6000B 3078 microscope (Buffalo Grove, IL, USA), and quantification was performed by ImageJ software.

### Transcript profiles analysis

Total RNA was extracted from the gastrocnemius muscles of male mice subjected to lower limb ischemia using TRIzol reagent (Invitrogen, CA, United States). The experimental groups included Sham (*n* = 4), Sham + Cal (*n* = 4), LLI (*n* = 4), and LLI + Cal (*n* = 3). cDNA libraries were constructed and sequenced on the Illumina NovaSeq 6000 platform at Gene Denovo Biotechnology Co. (Guangzhou, China). All subsequent analyses were conducted using high-quality clean data. Transcript abundances were quantified by calculating fragments per kilobase of transcript per million fragments mapped reads (FPKM) with RSEM software (Pertea et al. 2016). Differentially expressed genes (DEGs) were screened using DESeq2 software (Love et al. 2014), comparing groups with a fold change ≥ 1.5 and a *P*-value < 0.05. To explore the biological functions of the differentially expressed genes, Kyoto Encyclopedia of Genes and Genomes (KEGG) pathway enrichment analysis was performed using the clusterProfiler R package (Du et al. 2014).

### Statistical analysis

All statistical analyses were conducted using IBM SPSS Statistics version 26 and GraphPad Prism version 9.0. Continuous variables are presented as mean ± SD or as medians with interquartile range, while categorical variables were presented as percentages. Differences between groups were assessed using the Student’s t-test, Mann-Whitney U test, or Chi-square test. Group comparisons were performed using one-way ANOVA or two-way ANOVA. Survival rates and the incidence across groups were compared using the log-rank (Mantel-Cox) test, Breslow-Wilcoxon test, and χ² test. A *P*-value of less than 0.05 was considered statistically significant.

## Results

### General clinical characteristics of patients

A total of 104 patients diagnosed with aortic dissection were included in the study, among whom 11 (10.6%) presented with LLI. The patients were classified into two groups based on the presence or absence of lower extremity ischemia: the AD group, comprising patients with simple AD (*n* = 93), and the AD + LLI group, consisting of individuals with AD complicated by LLI (*n* = 11). A comparative analysis of the clinical data between these two groups was performed (Table 1). The duration of postoperative hospital stay and the incidence of hemodialysis post-surgery were significantly higher in the AD + LLI group compared to the AD group. No significant differences were observed in age, sex, or comorbidities between the AD and AD + LLI groups.

**Table 1:** Demographics and clinical characteristics of aortic dissection (AD) patients with and without lower limb ischemia (LLI)

	AD	AD + LLI	*P* value
	(*n* = 93)	(*n* = 11)	
Sex			
Male, n (%)	77 (82.8)	10 (91.0)	0.491
Female, n (%)	16 (17.2)	1 (9.0)	
Age	53.4 ± 13.2	50.9 ± 12.8	0.552
Height (cm)	169.4 ± 7.6	173.3 ± 8.6	0.432
Weight (kg)	72.5 ± 11.6	79.0 ± 14.6	0.462
Smoking Status, n (%)	12 (12.9)	3 (27.3)	0.200
Hypertension, n (%)	71 (76.3)	9 (81.8)	0.684
Systolic blood pressure (mmHg)	149.4 ± 23.8	146.7 ± 33.9	0.228
Diastolic blood pressure (mmHg)	82.1 ± 16.9	74.1 ± 15.5	0.472
Diabetes, n (%)	10 (10.8)	3 (27.3)	0.117
Hyperlipidemia, n (%)	4 (4.3)	1 (9.0)	0.483
Pericardial effusion, n (%)	8 (8.6)	3 (27.3)	0.052
Intraoperative indicators			
CPB (min)	93.9 ± 9.9	92.6 ± 10.6	0.698
ACC (min)	64.6 ± 3.9	63.8 ± 3.4	0.927
ACT (min)	22.5 ± 4.6	20.6 ± 5.4	0.212
hemodialysis, n (%)	6 (6.45)	3 (27.3)	**0.020**
Days of Hospitalization (d)	25.0 (21.0, 30.0)	38.0 (36.0, 45.0)	< **0.001**
Re-hospitalization, n (%)	16 (17.2)	4 (36.4)	0.127

Data are presented as mean ± SD, medians with interquartile ranges, or number (percentage), n (%). CPB: cardiopulmonary bypass; ACC: aortic clamping time; ACT: assisted circulation time. Continuous data were analyzed using either the t-test or non-parametric tests, while categorical data were assessed with the chi-square test. *P* < 0.05 was considered statistically different

Analysis of laboratory indicators between the two groups revealed that the mean leukocyte count, neutrophil count, and neutrophil percentage were significantly higher in the AD + LLI group compared to the AD group. Additionally, the AD + LLI group exhibited significantly elevated serum levels of aspartate aminotransferase (AST), creatinine, total cholesterol (TC), low-density lipoprotein (LDL), uric acid (UA), and creatine kinase (CK) relative to the AD group. Furthermore, the mean concentration of calcium ions was also significantly higher in the AD + LLI group (Table 2).

**Table 2:** Biological characteristics of patients

Variables	AD	AD + LLI	*P* value
	(*n* = 93)	(*n* = 11)	
Inflammation			
CRP (mg/L)	11.6 (4.8, 39.2)	7.8 (2.4, 55.1)	0.482
WBC (10^9^/L)	10.2 ± 3.3	13.0 ± 3.2	**0.011**
NE (10^9^/L)	8.6 ± 3.5	11.6 ± 3.0	**0.007**
NE (%)	83.9 (75.7, 90.1)	90.6 (86.4, 92.3)	**0.024**
LYM (10^9^/L)	0.96 (0.57, 1.28)	0.64 (0.47, 0.95)	0.125
LY (%)	9.8 (5.1, 15.9)	5.5 (3.5, 8.4)	**0.022**
MO (10^9^ /L)	0.5 (0.3, 0.7)	0.6 (0.3, 0.8)	0.583
MO (%)	5.6 ± 2.5	4.6 ± 2.5	0.221
Hepatic and Renal function			
ALT (U/L)	16.2 (11.0, 29.3)	21.7 (15.1, 52.1)	0.066
AST (U/L)	17.1 (14.3, 21.0)	26.4 (16.5, 67.8)	**0.036**
Cre (µmol/L)	80.2 (66.1, 97.8)	103.2 (71.4, 150.5)	**0.030**
UA (µmol/L)	364.1 ± 110.2	439.2 ± 114.4	**0.036**
CK (U/L)	125.1 ± 167.3	275.6 ± 214.4	**0.007**
Nutritional and Metabolic Marker			
GLU (mmol/L)	7.2 ± 2.2	8.0 ± 1.3	0.275
Hb (g/L)	127.0 (111.5, 138.5)	126.0 (112.0, 133.0)	0.779
TC (mmol/L)	4.2 ± 0.8	4.4 ± 1.0	0.365
TG (mmol/L)	1.09 (0.77, 1.43)	1.50 (1.19, 2.33)	**0.007**
HDL-C (mmol/L)	0.96 (0.82, 1.21)	0.97 (0.80, 1.25)	0.983
LDL-C (mmol/L)	2.6 ± 0.8	3.2 ± 1.1	**0.031**
Electrolyte indicators			
K^+^ (mmol/L)	4.65 ± 0.84	4.33 ± 0.78	0.232
Na^+^ (mmol/L)	139.7 ± 6.3	137.2 ± 6.6	0.219
Cl^−^ (mmol/L)	99.3 ± 6.0	99.9 ± 5.8	0.767
P^3+^ (mmol/L)	1.19 ± 0.26	1.27 ± 0.28	0.354
Ca^2+^ (mmol/L)	2.26 ± 0.23	2.51 ± 0.39	**0.002**

Data are expressed as mean ± SD, or medians with interquartile range. CRP, C-reactive protein; WBC, white blood cell count; NE, neutrophil count; NE (%), percentage of neutrophils; LYM, lymphocyte count; LY (%), lymphocyte percentage; MO, monocyte count; MO (%), monocyte percentage. ALT, alanine aminotransferase; AST, aspartate aminotransferase; Cre, creatinine; GLU, glucose; Hb, hemoglobin; TC, total cholesterol; TG, triglyceride; HDL-C, high-density lipoprotein cholesterol; LDL-C, low-density lipoprotein cholesterol; UA, uric acid; CK, creatine kinase. Continuous data were analyzed using either the t-test or non-parametric tests. Categorical data were assessed with the chi-square test. *P* < 0.05 was considered statistically different

### Elevated plasma SBDP145 levels in AD patients with lower limb ischemia

**Fig. 1:**
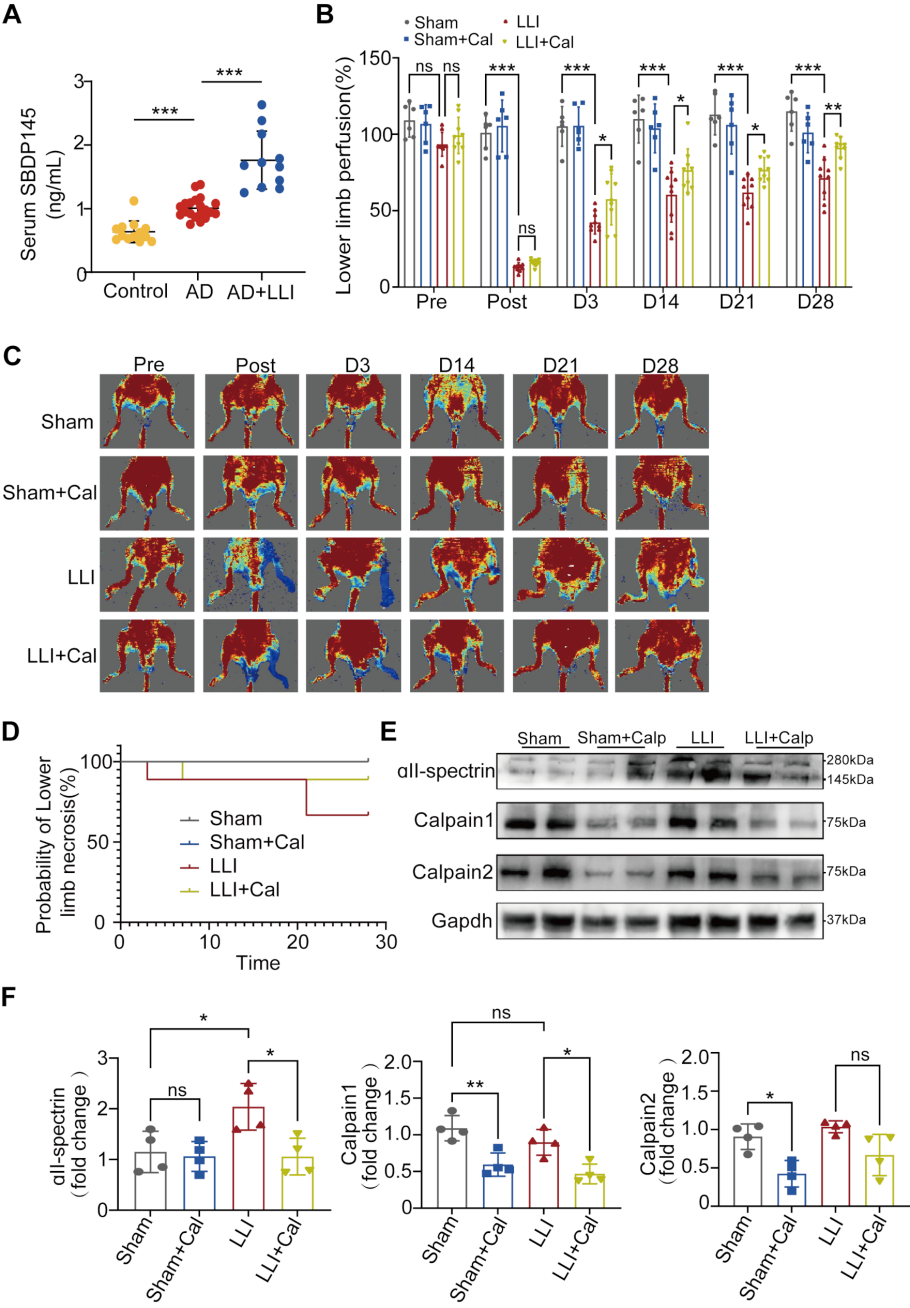
Inhibition of Calpain enhanced blood flow recovery in a mouse model of LLI. (**A**) Plasma concentrations of SBDP145 among three groups: Control (*n* = 13), Aortic dissection (AD) without LLI (*n* = 20), and AD patients with LLI (*n* = 11). Data are presented as the mean ± SD and analyzed using One-way ANOVA. (**B**) Quantitative analysis of blood perfusion was conducted using two-way ANOVA. Sham and Sham + Cal groups: *n* = 6 per group; LLI and LLI + Cal groups: *n* = 9 per group. (**C**) Blood perfusion images for the ischemic hindlimbs were obtained pre-surgery and on days 0, 3, 14, 21, and 28 post-surgery. (**D**) The probability of lower limb necrosis. (**E**) The expression levels of ɑII-Spectrin, Calpain1, and Calpain2 in the gastrocnemius muscle were examined by Western blotting across all groups. (**F**) Quantification of Western blotting for ɑII-Spectrin, Calpain1, and Calpain2 was performed with *n* = 4 for the pre group. *, *P* < 0.05; **, *P* < 0.01; ***, *P* < 0.001; ns, no significant difference

These findings indicate that the calcium ion concentration significantly influences the occurrence of LLI in patients with AD. Calpain, a calcium-dependent proteolytic enzyme, is implicated in the pathology of various diseases (Muniappan et al. 2021). Plasma concentrations of SBDP145, a specific indicator of Calpain activity, were quantified using ELISA. Plasma samples were collected from AD patients with or without LLI, and from healthy medical examiners. The plasma levels of SBDP145 in the AD-LLI group were significantly higher than those in AD and healthy groups (Fig. 1A).

### Calpain inhibition promoted blood flow recovery in lower limb ischemia mouse model

To investigate the role of Calpain in LLI, we established a mouse model of lower limb ischemia and administered the Calpain inhibitor Calpeptin as an intervention. Laser Doppler images revealed that there was virtually no blood flow in the left hindlimbs of both LLI and LLI + Cal group, with blood flow sharply dropping to less than 20% of that on the normal side. Blood flow perfusion in the ischemic limb began to recover gradually after day 3 post-surgery. Notably, Calpain inhibition enhanced blood flow recovery following left femoral artery ligation surgery. By days 21 and 28, perfusion had recovered to nearly 70% and 90%, respectively (Fig. 1B and C). Additionally, the incidence of necrosis was lower in the LLI + Cal group compared to the LLI group, with the onset of necrosis occurring later (Fig. 1D). By day 28 post-surgery, no mice died in any of the groups. Furthermore, the 145 kD fragment of ɑII-spectrin, which is indicative of Calpain activity, was increased in the LLI group, while the levels were significantly inhibited in the LLI + Cal group. Calpeptin significantly decreased the expression levels of Calpain-1 and Calpain-2 in both the Sham + Cal and LLI + Cal groups (Fig. 1E and F). These results suggest that Calpain plays a critical role in blood flow recovery under ischemic conditions.

### Calpain inhibition promoted vascular regeneration and reduced inflammation in ischemic tissue

**Fig. 2:**
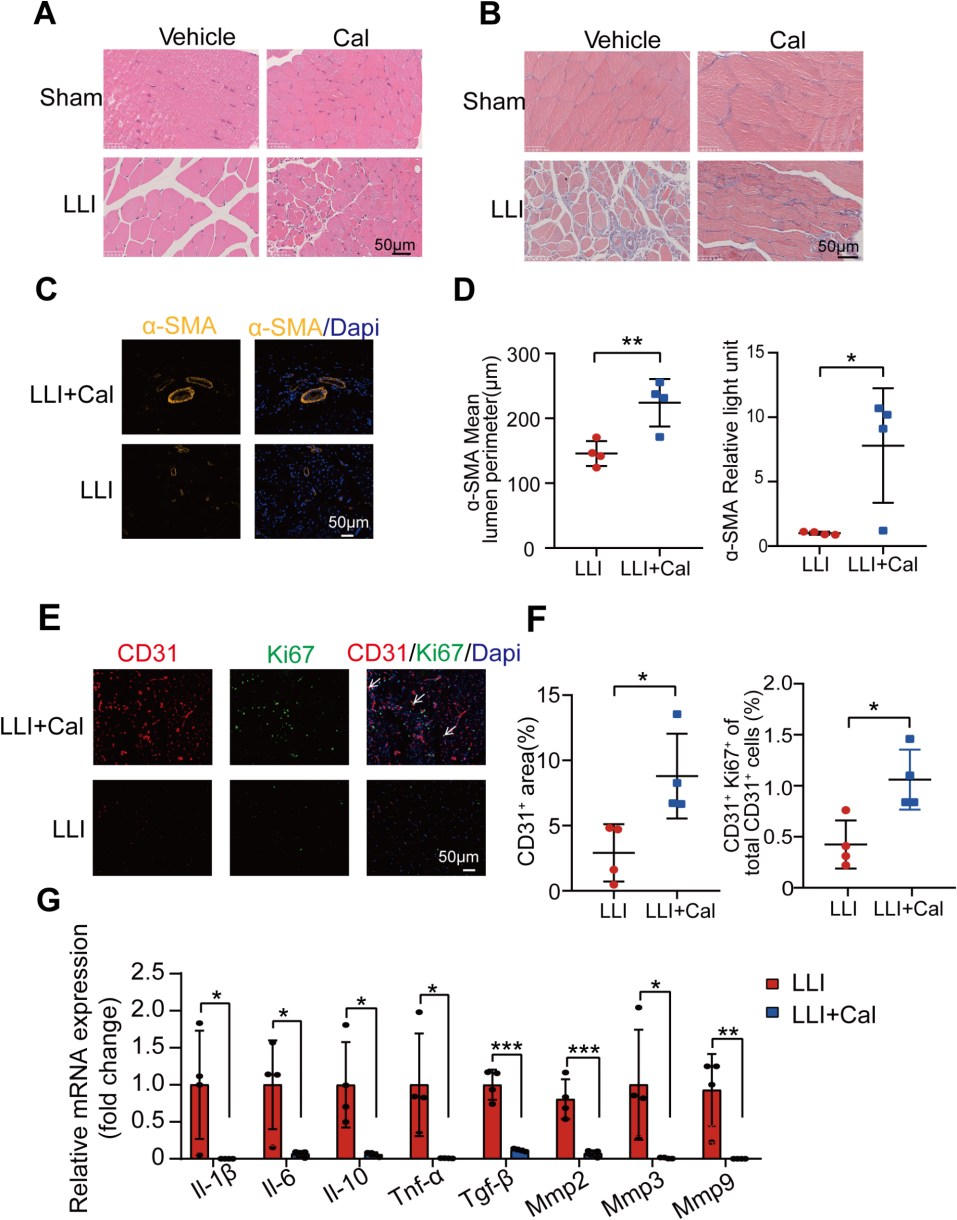
Inhibition of calpain promoted angiogenesis and reduced inflammatory response in the ischemic hindlimb. (**A**) Representative HE staining images of gastrocnemius muscles on day 28 across all groups. (**B**) Representative Masson staining images indicating collagen fiber deposition. (**C**) Representative immunofluorescence (IF) staining images of α-SMA in both the LLI and LLI + Cal groups. (**D**) Statistical analysis of the α-SMA^+^ lumen perimeter and fluorescence density. *n* = 4 per group. (**E**) Representative IF images of CD31 and Ki67. (**F**) Statistical analysis of the percentage of CD31^+^ area and the proportion of CD31^+^Ki67^+^ cells. *n* = 4 per group. (**G**) Statistical analysis of pro-inflammatory and anti-inflammatory gene expression in the LLI and LLI + Cal groups on Day 7. *n* = 4 per group. Scale bar: 50 μm. One-way ANOVA was performed to compare the variance among groups. *, *P* < 0.05; **, *P* < 0.01; ***, *P* < 0.001

We collected gastrocnemius muscle samples from the operated side of mice 28 days post-surgery for histological analysis. HE and Masson staining revealed numerous cavities and significant collagen fiber deposition in the LLI group, indicating pronounced necrosis and fibrosis in this tissue region. In contrast, the LLI + Cal group displayed a more densely arranged structure, with fewer cavities, reduced tissue necrosis, and significantly lower collagen fiber deposition (Fig. 2A and B). Furthermore, we performed immunofluorescence staining on the gastrocnemius muscles to assess vascular regeneration. The α-SMA immunofluorescence images indicated that the average vessel length in the LLI + Cal group was greater than that in the LLI group, accompanied by an increase in signal intensity. Additionally, CD31/Ki67 double-label immunofluorescence staining demonstrated that the CD31 area in the LLI + Cal group exceeded that of the LLI group, along with an elevated Ki67 positive rate in the LLI + Cal group (Fig. 2C-F).

In the early stages, inflammation is associated with ischemic tissue damage (Bassiouni et al. 2021; Ma et al. 2024). We collected gastrocnemius muscle samples from mice on the seventh day post-surgery and quantitatively assessed inflammatory factors using qPCR. The results revealed that the expression levels of Il-1β, Il-6, Il-10, Tnf-α, Tgf-β1, Mmp2, Mmp3, and Mmp9 in the LLI + Cal group were significantly lower than those in the LLI group, suggesting that Calpain inhibition mitigated the inflammatory response (Fig. 2G).

### Endogenous specific inhibitor promoted blood flow recovery

**Fig. 3:**
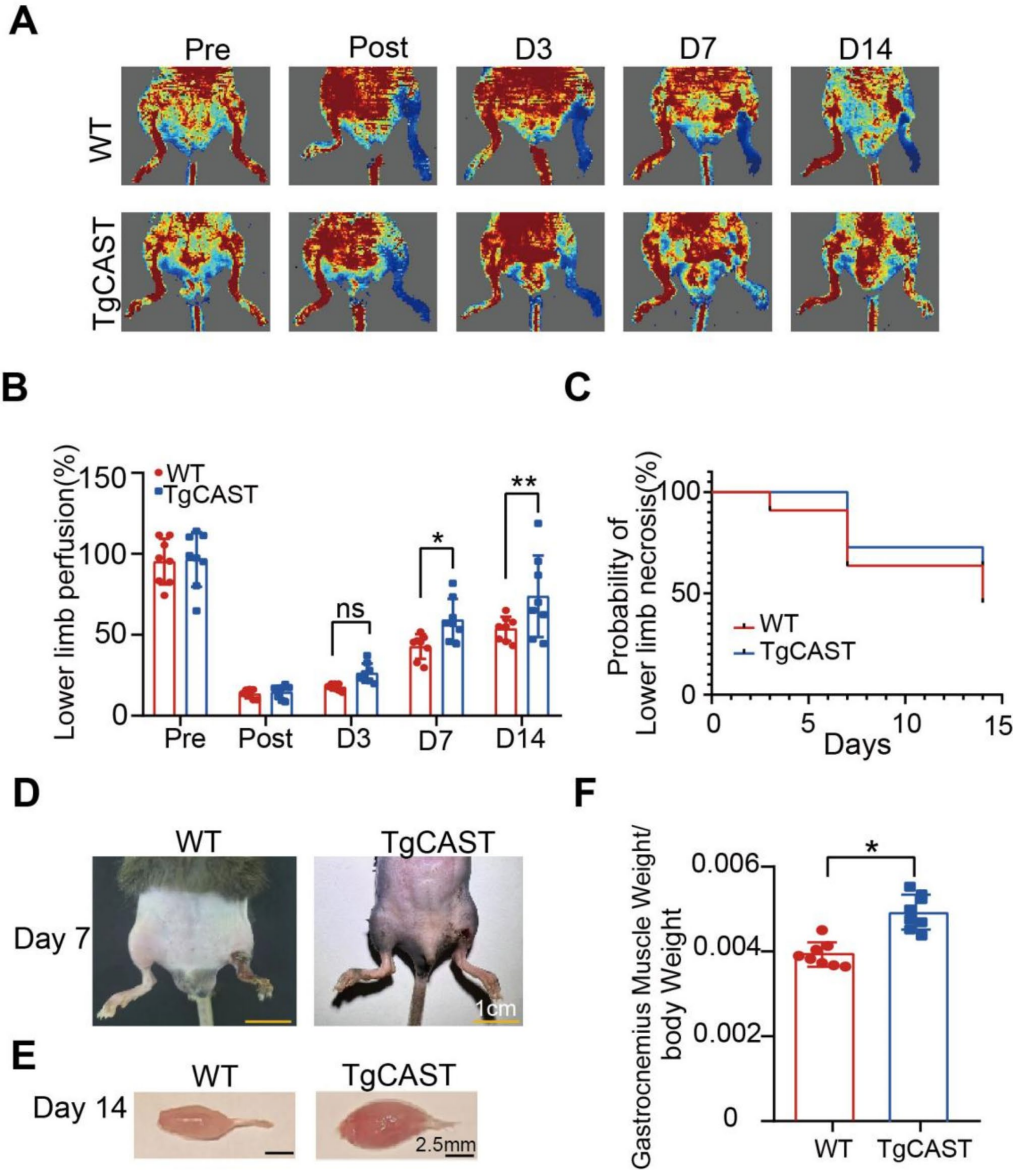
Endogenous specific inhibitor promoted blood flow recovery. Transgenic mice overexpressing calpastatin (TgCAST) and their wild-type (WT) littermates were subjected to lower limb ischemia. (**A**) Blood perfusion images for the ischemic hindlimbs were obtained pre-surgery and on days 0, 3, 7 and 14 post-surgery. (**B**) Statistical analysis of the blood perfusion ratio between the TgCAST and WT groups. *n* = 8 per group. (**C**) The probability of lower limb necrosis. *n* = 8 per group. (**D**) Representative images of lower limb necrosis in mice at 7 days. (**E**) Representative images of gastrocnemius muscle at postoperative day 14. (**F**) The gastrocnemius to body weight ratio in mice. *n* = 8 per group. The one-way analysis of variance was performed to compare data between groups. *, *P* < 0.05; **, *P* < 0.01; ns, no significant difference

Calpastatin is a specific endogenous inhibitor of Calpain-1 and Calpain-2 (Nian and Ma 2021; Zheng et al. 2021). To further elucidate the role of Calpain, transgenic mice overexpressing calpastatin (TgCAST) and their wild-type (WT) littermates were subjected to lower limb ischemia. We monitored blood flow for fourteen days following femoral artery ligation. TgCAST mice demonstrated significantly improved blood flow recovery compared to WT mice on both the seventh and fourteenth days post-surgery, along with a lower incidence of toe necrosis and a delayed onset of necrosis (Fig. 3A-D). On day 14 post-surgery, the ratio of gastrocnemius muscle to body weight in the TgCAST group was greater than that in the WT group (Fig. 3E-F). These findings support the role of Calpain inhibitors in promoting angiogenesis and protecting muscle in LLI models. Thus, both the Calpain inhibitor Calpeptin and the overexpression of endogenous Calpastatin effectively enhance recovery from lower limb ischemia.

### Transcriptome profiling reveals the mechanism of Calpain inhibition for lower limb ischemia protection

To summarize the transcriptomic changes associated with Calpain inhibition, mRNA sequencing was performed on mice that underwent lower limb ischemia surgery, both with and without Calpeptin treatment. Volcano plots revealed the upregulated and downregulated genes in the Sham versus LLI and LLI versus LLI + Cal groups (Fig. S2A and B). The differentially expressed genes (DEGs) were involved in regulating ECM-receptor interactions, focal adhesion, the PI3K-Akt signaling pathway and oxidative phosphorylation (Fig. S2C and D).

**Fig. 4:**
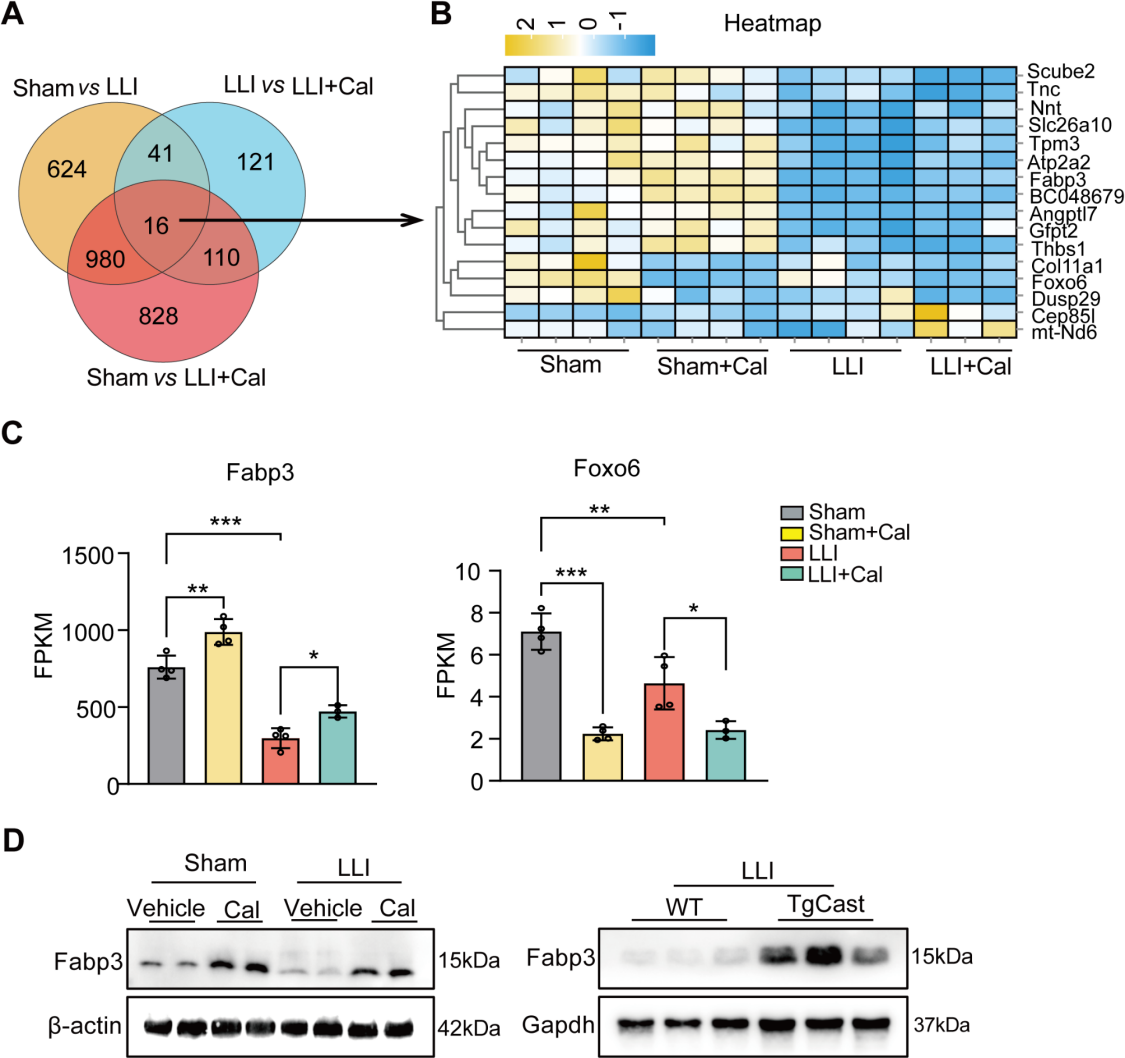
RNA-seq analysis for pharmacological inhibition of Calpain. (**A**) A Venn diagram analysis across four groups. (**B**) A heatmap depicting the expression of 16 genes among four groups. (**C**) FPKM values for Fabp3 and Foxo6 across four groups. Sham, *n* = 4; Sham + Cal, *n* = 4; LLI, *n* = 4; LLI + Cal, *n* = 3. Data are presented as the mean ± SD. *P* values were calculated by One-way ANOVA, followed by Tukey’s multiple comparison. (**D**) Representative Western blot results for Fabp3 were presented, with β-actin or Gapdh serving as the loading control. *, *P* < 0.05; **, *P* < 0.01; ***, *P* < 0.001

A Venn diagram pinpointed DEGs specifically linked to Calpain inhibition, including Fabp3, Foxo6, mt-Nd6 Cep85l, Dusp29, Col11a1, Thbs1, Gfpt2, Angptl7, BC048679, Atp2a2, Tpm3, Slc26a10, Nnt, Tnc, Scube2 (Fig. 4A). Heatmap analysis and FPKM values indicated a significant downregulation of Fabp3 in the LLI group, with an increase expression following Calpain inhibition. Conversely, Foxo6 exhibited opposite trends, while other genes did not show significant differences (Fig. 4B and C, Fig. S3). Notably, Fabp3 emerged as the most abundantly expressed differentially regulated gene in this RNA-seq dataset. Western blot analysis further confirmed that Calpain regulation of Fabp3 plays a critical role in recovery from LLI (Fig. 4D). The injection of the Fabp3 overexpression plasmid into the ischemic hindlimb of mice did not enhance blood flow recovery; however, it did improve the preservation rate of the lower limb in these mice (Fig. S4). These results suggest that Fabp3 contributes to the protection of muscle tissue and mitigates lower limb necrosis in treatments involving Calpain inhibitors.

### Pharmacologic blockade of Calpain protects mice from BAPN-Induced AD

**Fig. 5:**
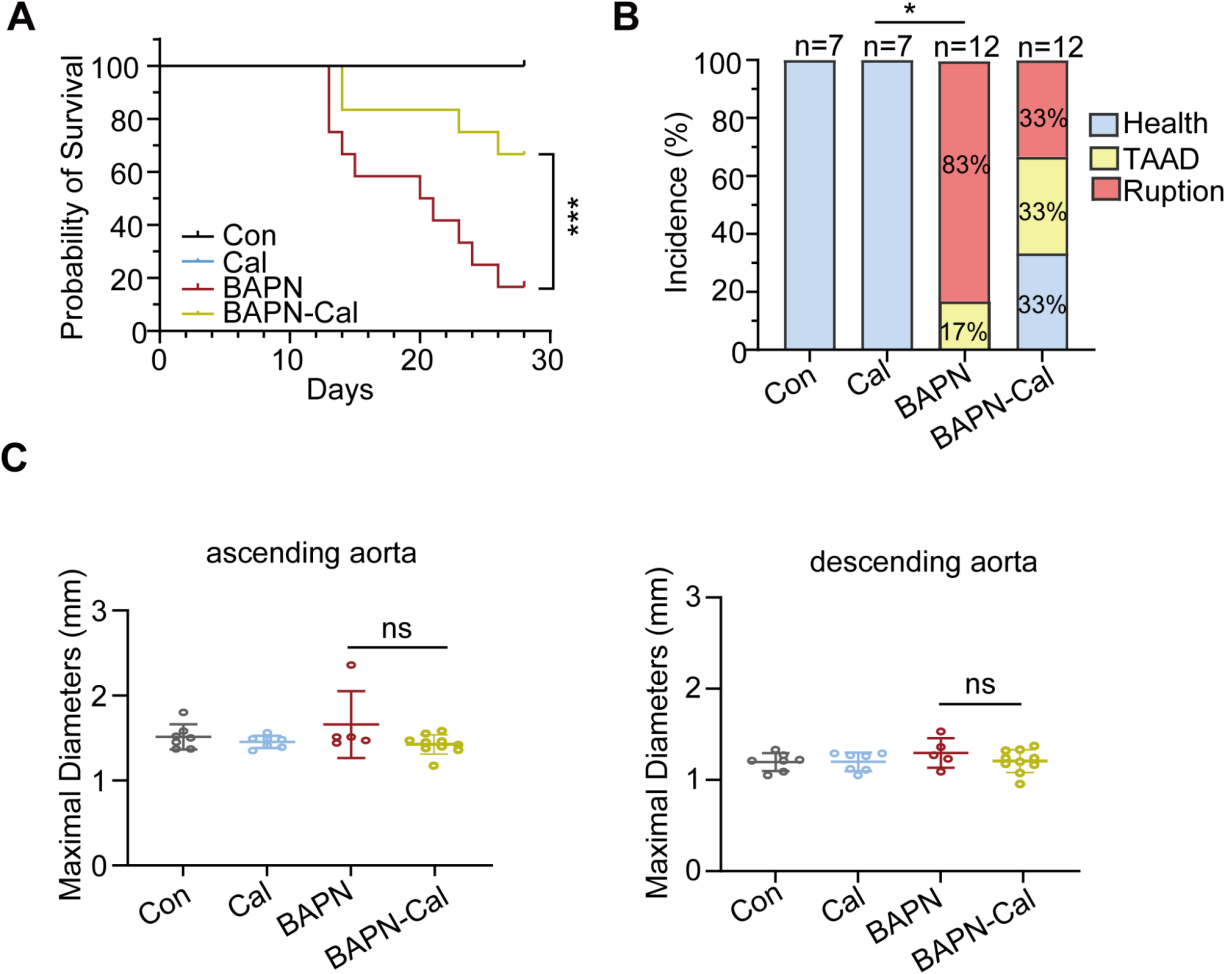
Inhibition of Calpain reduced BAPN-induced AD formation in male mice. Pharmacological inhibition of Calpain using Calpeptin. Con, *n* = 7; Cal, *n* = 7; BAPN, *n* = 12; BAPN-Cal, *n* = 12. (**A**) The survival rate was estimated using the Kaplan-Meier method and compared via the log-rank test. (**B**) The incidence of thoracic aortic aneurysm and dissection (TAAD). Statistical significance was by chi-square test. (**C**) In situ measurements of maximal diameters of ascending and descending thoracic regions in mice administered vehicle vs. BAPN for 3 weeks. Con, *n* = 7; Cal, *n* = 7; BAPN, *n* = 5; BAPN-Cal, *n* = 10. Statistical significances were determined by One-way ANOVA with Tukey’s multiple comparison. *, *P* < 0.05; ***, *P* < 0.001; ns, no significant difference

To investigate the role of Calpain inhibition in AD, a mouse model of aortic dissection was established using BAPN in drinking water, followed by treatment with the Calpain inhibitor Calpeptin. The survival rates and incidence of AD indicated that Calpain inhibition provided protection against the formation and progression of AD; however, no significant changes were observed in aortic diameter (Fig. 5). These findings suggest that Calpain inhibition not only effectively prevents the formation of AD but may also enhance the recovery of blood flow in cases of LLI.

## Discussion

AD could be complicated by malperfusion affecting the coronary, cerebral, mesenteric, renal, and lower extremity systems, which complicates clinical management and therapeutic strategies. In severe cases of malperfusion, the mortality rate due to limb ischemia can even surpass that of dissection rupture (Mur Villacampa 1987). In this study, we found that patients with AD and LLI exhibited significantly elevated levels of leukocyte cells, AST, TC, LDL, UA, CK, and calcium ions. Furthermore, we recognized Calpain as a potential therapeutic target for AD combined with LLI. Through animal experiments, we demonstrated for the first time that inhibiting Calpain in mice can facilitate the restoration of blood flow to the hind limbs of ischemic mice and reduce the incidence of residual limbs by inhibiting the ischemia-induced inflammatory response, and promoting vascular regeneration. Importantly, we discovered that Calpain inhibition leads to an increase in Fabp3 levels, suggesting that the therapeutic effects of Calpain inhibition may be linked to the enhancement of the fatty acid metabolism pathway.

This study analyzed data from 104 patients diagnosed with AD, of whom 10.6% presented with LLI, aligning with existing literature (Jaffar-Karballai et al. 2021). Notably, the incidence of AD accompanied by LLI was found to be higher in men than in women, accounting for over 80% of cases. We distinguished between the AD and AD + LLI groups, and found that patients in the AD + LLI group exhibited significantly elevated levels of leukocyte cells, AST, TC, LDL, UA, CK, and calcium ions. Additionally, patients with LLI showed increased levels of AST and Cre compared to the control group. Furthermore, the white blood cell count, neutrophil count, neutrophil proportion, and lymphocyte count in patients with LLI were all significantly higher than those in the control group, indicating a more pronounced inflammatory response in these patients.

To investigate the therapeutic effects of Calpain inhibition on lower limb ischemic diseases, we developed a mouse model of lower limb ischemia. The administration of the exogenous drug inhibitor Calpeptin, along with the endogenous inhibitor Calpastatin, significantly enhances blood perfusion recovery in the ischemic limbs of mice, protects muscle cells, reduces hindlimb necrosis, and markedly improves the limb preservation rate.

An increasing number of studies have demonstrated that Calpain plays a significant role in apoptosis and fibrosis. The activation of Calpain in mitochondria results in decreased activity of mitochondrial complex I, a reduction in the mitochondrial membrane potential of myoblasts, and the induction of myoblast apoptosis (Zeng et al. 2022). Overexpression of calpastatin can mitigate fibrosis in the aorta and surrounding tissues, which is accompanied by a decrease in the activity of matrix metalloproteinases (Mmps) (Letavernier et al. 2008). The activation of the epidermal growth factor receptor (EGFR) leads to an increase in intracellular calcium levels and the activation of mitogen-activated protein kinase (MAPK), which subsequently activates Calpain. This EGFR-dependent activation of Calpain is implicated in signaling pathways that regulate the synthesis of extracellular matrix (ECM) proteins, including the modulation of transforming growth factor-β (TGF-β) expression (Creemers and Pinto 2011). In this experiment, following calpain inhibition, the expression of TGF-β1, Mmp2, Mmp3, and Mmp9 in gastrocnemius muscle tissue was significantly reduced, thereby inhibiting fibrosis in the muscle tissue.

In the context of hypercholesterolemia and chronic myocardial ischemia, Calpain inhibition has been shown to reduce collagen deposition in both ischemic and non-ischemic myocardial tissues. This reduction in collagen content is associated with the downregulation of the Jak/STAT/MCP-1 signaling pathway (Potz et al. 2022). In a rat stroke model, the administration of a Calpain inhibitor mitigated the inflammatory response by suppressing the PARP-NF-κB pathway, which in turn reduced damage to the neurovascular unit and improved neurological function (Yan et al. 2024). Our findings indicate that Calpeptin effectively inhibits the expression of several pro-inflammatory factors, including IL-1β, IL-6, and TNF-α, while also promoting angiogenesis in mouse muscle tissue. Previous studies have established that Calpain is activated in models of myocardial ischemia-reperfusion injury, exacerbating damage by inhibiting vascular regeneration (Perrin et al. 2003). Consequently, Calpain plays a critical role in vascular ischemic lesions.

The molecular mechanisms by which Calpain inhibits protective effects against lower limb ischemia demonstrated that Fabp3 was regulated by Calpain and exhibited significant higher expression. Fabp3, a member of the lipid chaperone family, also known as heart-type fatty acid binding protein (H-FABP), plays a critical role in the solubilization, transport, and metabolism of cellular fatty acids. It is integral to lipid metabolism, gene regulation, and cell signaling, and is essential for cell proliferation and differentiation (Guo et al. 2023). The deficiency in Fabp3 leads to reduced fatty acid oxidation (FAO), increased glycolysis, and abnormal lipid accumulation, thereby exacerbating cardiac hypertrophy and dysfunction induced by aortic dissection (Zhuang et al. 2021). Dietary fatty acids enhance the expression of Fabp-4 and Fabp-3, stimulating the secretion of key angiogenic factors such as VEGF and angiopoietin-like protein 4 (ANGPTL4), which in turn induces angiogenesis (Basak et al. 2013). In an in vivo model of anti-angiogenic therapy, treatment with bevacizumab resulted in the upregulation of Fabp3 and Fabp7, both of which were induced through HIF-1α-dependent mechanisms. Under hypoxic conditions, a significant accumulation of lipid droplets (LD) is influenced by a reduction in the expression of endogenous Fabp3, Fabp7, or lipophilin. This reduction significantly impacts LD formation under hypoxic conditions and contributes to the growth and survival of cells in such an environment (Bensaad et al. 2014). However, Fabp3 expression is elevated in the thickened adventitia of patients with idiopathic arteritis, and the overexpression of Fabp3 may promote vascular fibrosis by enhancing fatty acid oxidation in aortic fibroblasts (Wu et al. 2022). The conflicting results may be attributed to variations in disease models and tissue specificity.

This study found that Calpain inhibition not only effectively prevented the formation of AD but also enhanced blood flow recovery in cases of lower limb ischemia. This improvement was mediated by an increase in Fabp3 expression following Calpain inhibition. The precise molecular mechanisms underlying these effects require further investigation.

## Conclusion

The inhibition of Calpain has the potential to decrease the incidence of AD and enhance the restoration of blood flow perfusion in ischemic lower extremities. This effect may be attributed to the upregulation of Fabp3. These findings further underscore the potential application value of targeted interventions against Calpain as an emerging therapeutic strategy for cardiovascular disease.

## Electronic supplementary material

Below is the link to the electronic supplementary material.

Supplementary Material 1

## Data Availability

No datasets were generated or analysed during the current study.

## Abbreviations

AD: Aortic dissection
LLI: Lower limb ischemia
CTA: Computed tomography angiography
WT: Wild-type
H&E: Hematoxylin and eosin
ALT: Alanine aminotransferase
AST: Aspartate aminotransferase
CRE: Creatinine
CPB: Cardiopulmonary bypass
ACC: Aortic clamping time
ACT: Assisted circulation time
CRP: C-reactive protein
WBC: White blood cell count
NE: Neutrophil count
LYM: Lymphocyte count
MO: Monocyte count
GLU: Glucose
Hb: Hemoglobin
TC: Total cholesterol
TG: Triglyceride
HDL-C: High-density lipoprotein cholesterol
LDL-C: Low-density lipoprotein cholesterol
UA: Uric acid
CK: Creatine kinase
TG: Triglyceride
IL: Interleukin
Tnf: Tumor necrosis factor
Tgf: Transforming growth factor
MMP: Matrix metalloproteinase
AngII: Angiotensin II
EGFR: Epidermal growth factor receptor
MAPK: Mitogen-activated protein kinase
ECM: Extracellular matrix
H-FABP: Heart-type fatty acid binding protein
FAO: Fatty acid oxidation
ANGPTL4: Angiopoietin-like protein 4
LD: Lipid droplets
