# Correction: Calpain inhibition as a novel therapeutic strategy for aortic dissection with acute lower extremity ischemia

**DOI:** 10.1186/s10020-026-01508-2

**Published:** 2026-05-25

**Authors:** Qiwen Tan, Xiaokang Wang, Wanchuang Xu, Kun Song, Yifan Xiong, Zhentong Jiang, Jingjing Li, Yunsheng Yu, Wenxue Ye, Zhenya Shen, Xiaomei Teng

**Affiliations:** 1 Department of Cardiovascular Surgery, First Affiliated Hospital of Soochow University, 899 Pinghai Road, Suzhou, 215006 China; 2 Suzhou Medical College, Soochow University, 199 Renai Road, Suzhou, 215123 China; 3 Institute for Cardiovascular Science, Soochow University, 178 Ganjiang Road, Suzhou, 215006 China

**Correction: Molecular Medicine 31, 144 (2025)**

**https://doi.org/10.1186/s10020-025-01212-7**

In this article (Tan et al. 2026), Fig. 1 appeared incorrectly and have now been corrected in the original publication. For completeness and transparency, both correct and incorrect versions are displayed below.

The original article has been corrected.

Incorrect Fig. 1.

**Fig. 1:**
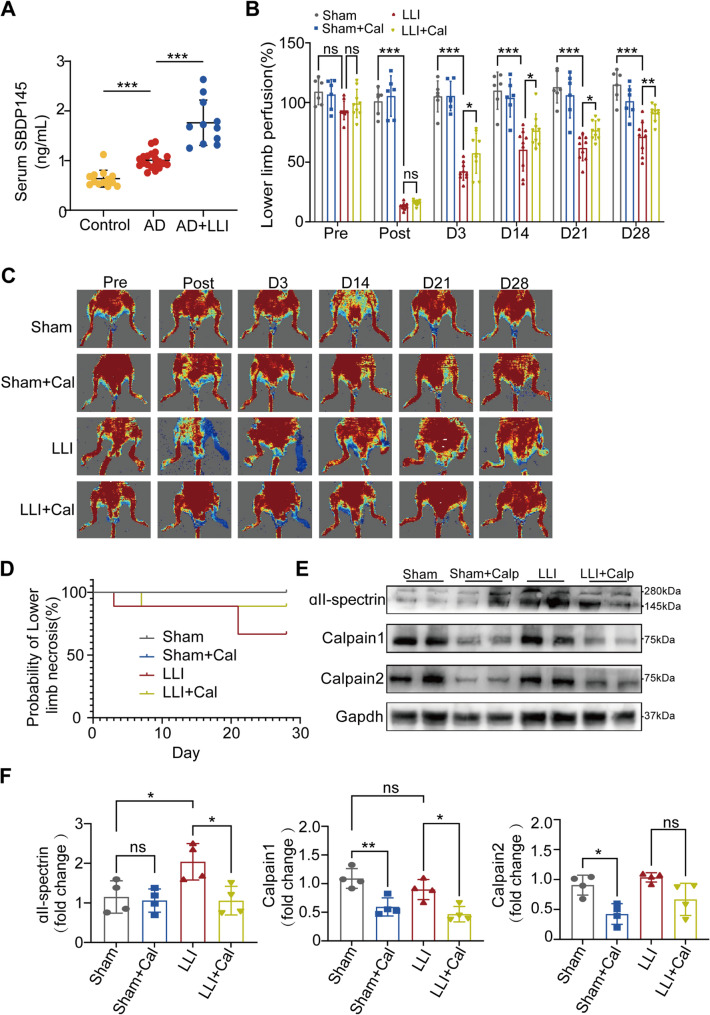
Inhibition of Calpain enhanced blood flow recovery in a mouse model of LLI. **A** Plasma concentrations of SBDP145 among three groups: Control (*n* = 13), Aortic dissection (AD) without LLI (*n* = 20), and AD patients with LLI (*n* = 11). Data are presented as the mean ± SD and analyzed using One-way. ANOVA. **B** Quantitative analysis of blood perfusion was conducted using two-way ANOVA. Sham and Sham + Cal groups: *n* = 6 per group; LLI and LLI + Cal groups: *n* = 9 per group. **C** Blood perfusion images for the ischemic hindlimbs were obtained pre-surgery and on days 0, 3, 14, 21, and 28 postsurgery. **D** The probability of lower limb necrosis. **E** The expression levels of ɑII-Spectrin, Calpain1, and Calpain2 in the gastrocnemius muscle were examined by Western blotting across all groups. **F** Quantification of Western blotting for ɑII-Spectrin, Calpain1, and Calpain2 was performed with *n* = 4 for the pre group. *, *P* < 0.05; **, *P* < 0.01; ***, *P* < 0.001; ns, no significant difference.

Correct Fig. 1.

**Fig. 2:**
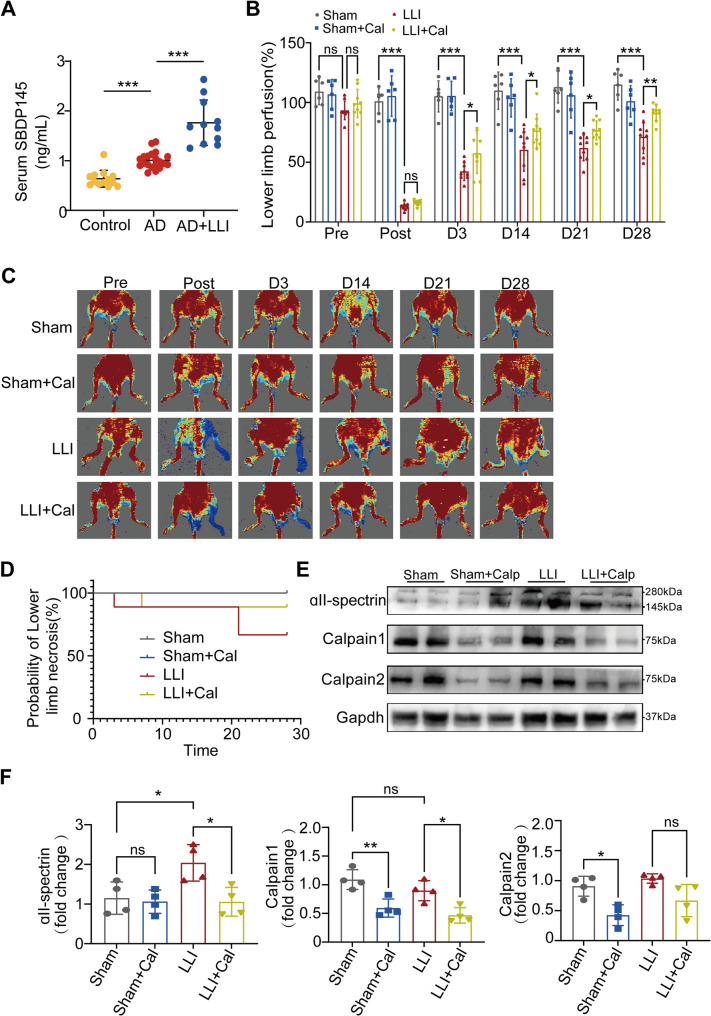
Inhibition of Calpain enhanced blood flow recovery in a mouse model of LLI. **A** Plasma concentrations of SBDP145 among three groups: Control (*n* = 13), Aortic dissection (AD) without LLI (*n* = 20), and AD patients with LLI (*n* = 11). Data are presented as the mean ± SD and analyzed using One-way. ANOVA. **B** Quantitative analysis of blood perfusion was conducted using two-way ANOVA. Sham and Sham + Cal groups: *n* = 6 per group; LLI and LLI + Cal groups: *n* = 9 per group. **C** Blood perfusion images for the ischemic hindlimbs were obtained pre-surgery and on days 0, 3, 14, 21, and 28 postsurgery. **D** The probability of lower limb necrosis. **E** The expression levels of ɑII-Spectrin, Calpain1, and Calpain2 in the gastrocnemius muscle were examined by Western blotting across all groups. **F** Quantification of Western blotting for ɑII-Spectrin, Calpain1, and Calpain2 was performed with *n* = 4 for the pre group. *, *P* < 0.05; **, *P* < 0.01; ***, *P* < 0.001; ns, no significant difference
