# Supplementary material for: Calpain inhibition as a novel therapeutic strategy for aortic dissection with acute lower extremity ischemia
Source: Mol Med. 2025 Apr 21;31:144. doi: 10.1186/s10020-025-01212-7 (PMC12013106; doi:10.1186/s10020-025-01212-7)
Supplement: Supplementary file 1 — Supplementary Material 1 [file 10020_2025_1212_MOESM1_ESM.pdf]

Supplementary material

Title: Calpain Inhibition as a Novel Therapeutic Strategy for Aortic Dissection with Acute Lower Extremity Ischemia

Authors

Qiwen Tan†, Xiaokang Wang†, Wanchuang Xu†, Kun Song†, Yifan Xiong, Zhentong Jiang, Jingjing Li, Yunsheng Yu*， Wenxue Ye*， Zhenya Shen, Xiaomei Teng*

This PDF file includes:

Method

Supplemental figures: Fig. S1 to S4

Supplemental tables: Tables S1and S2

Method

Plasmid Transfection in vivo

Plasmid DNA was transfected into the gastrocnemius muscle of mice using an in vivo DNA transfection reagent (in vivo-jetPEI®)(Polyplus-transfection, Illkirch, France). The pECMV or pECMV-Fabp3 plasmid was diluted to half the injection volume in 5% glucose (final concentration) using a 10% glucose stock solution (in vivo-jetPEI®, 201-10G and 201-50G). Subsequently, the in vivo-jetPEI® reagent was also diluted to half the injection volume in 5% glucose (final concentration) using the same 10% glucose stock solution and sterile water. The diluted in vivo-jetPEI® reagent was then combined with the diluted nucleic acid all at once. The mixture was incubated for 15 minutes at room temperature. Following this, 50 μL of the complexes, equilibrated at room temperature, were injected into the gastrocnemius muscle of the mice. Mice received injections three times a week.

Supplemental figures and legends

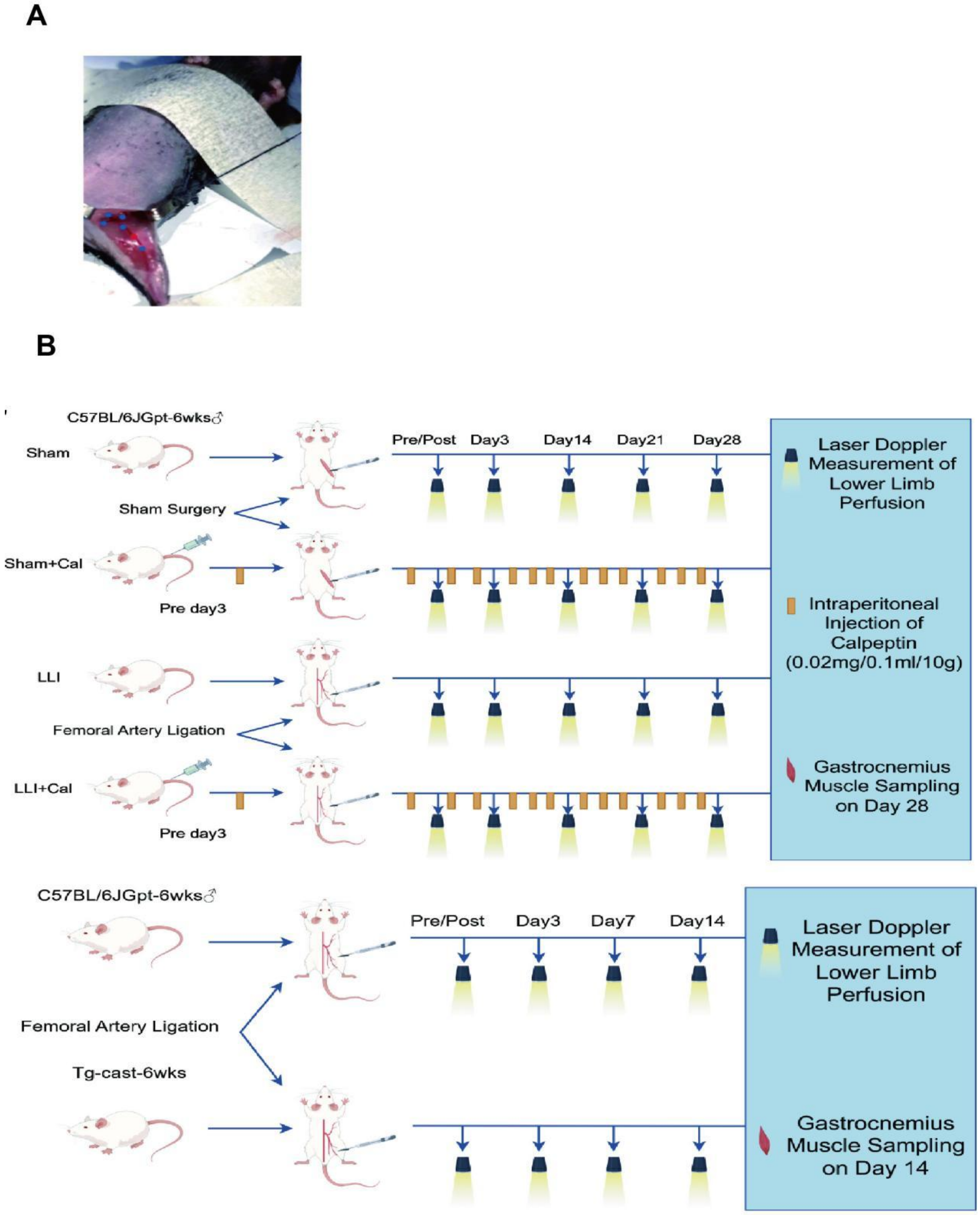

Fig.S1.The establishment and experimental design of a lower limb ischemia model in mice. A. A real image of the surgical procedure for inducing lower limb ischemia in mice, with the blue dot indicating the ligation site and the red dot marking the site of blood vessel transection. B. A schematic flow chart that illustrates the process of calpeptin injection, followed by Doppler measurement of blood perfusion in the lower limbs of mice at various time points. This chart was created using Figdraw.

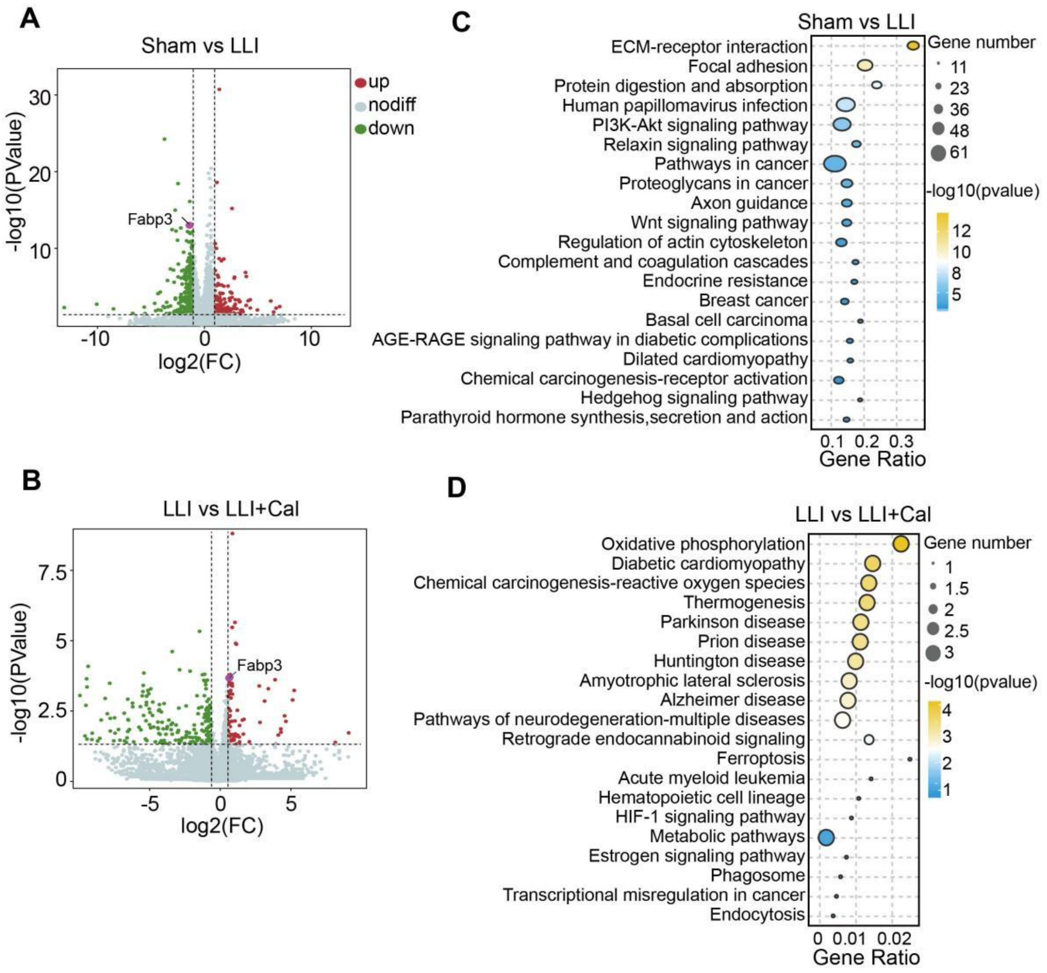

Fig.S2. RNA-seq for Calpain inhibition. Screening of differentially expressed genes (DEGs) from mice treated with lower limb ischemia operation, with or without Calpeptin. (A and B) Volcano plots of DEGs in the gastrocnemius muscles from mice, comparing groups with a fold change ⩾ 1.5 and P-value < 0.05. Kyoto Encyclopedia of Genes and Genomes (KEGG) pathway classifications for DEGs between Sham and LLI groups (C) or between LLI and LLI-Calpeptin groups (D).

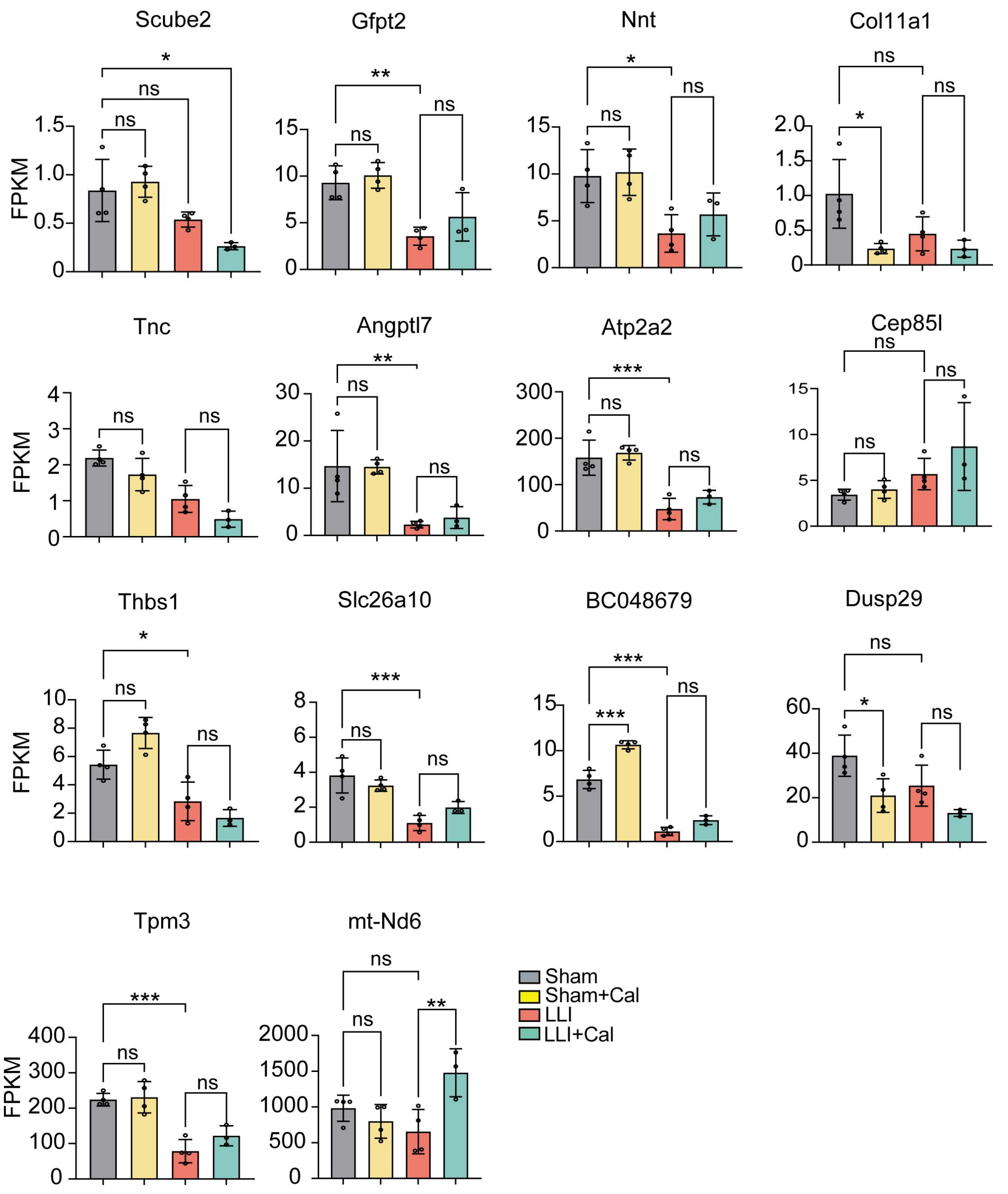

Fig.S3. FPKM values for 14 genes across four groups. Sham, n = 4; Sham + Cal, n = 4; LLI, n = 4; LLI + Cal, n = 3. Data are presented as the mean ± SD. P values were calculated by One-way ANOVA, followed by Tukey's multiple comparison. *, $P < 0.05$; **, $P < 0.01$; ***, $P < 0.001$; ns, no significant difference.

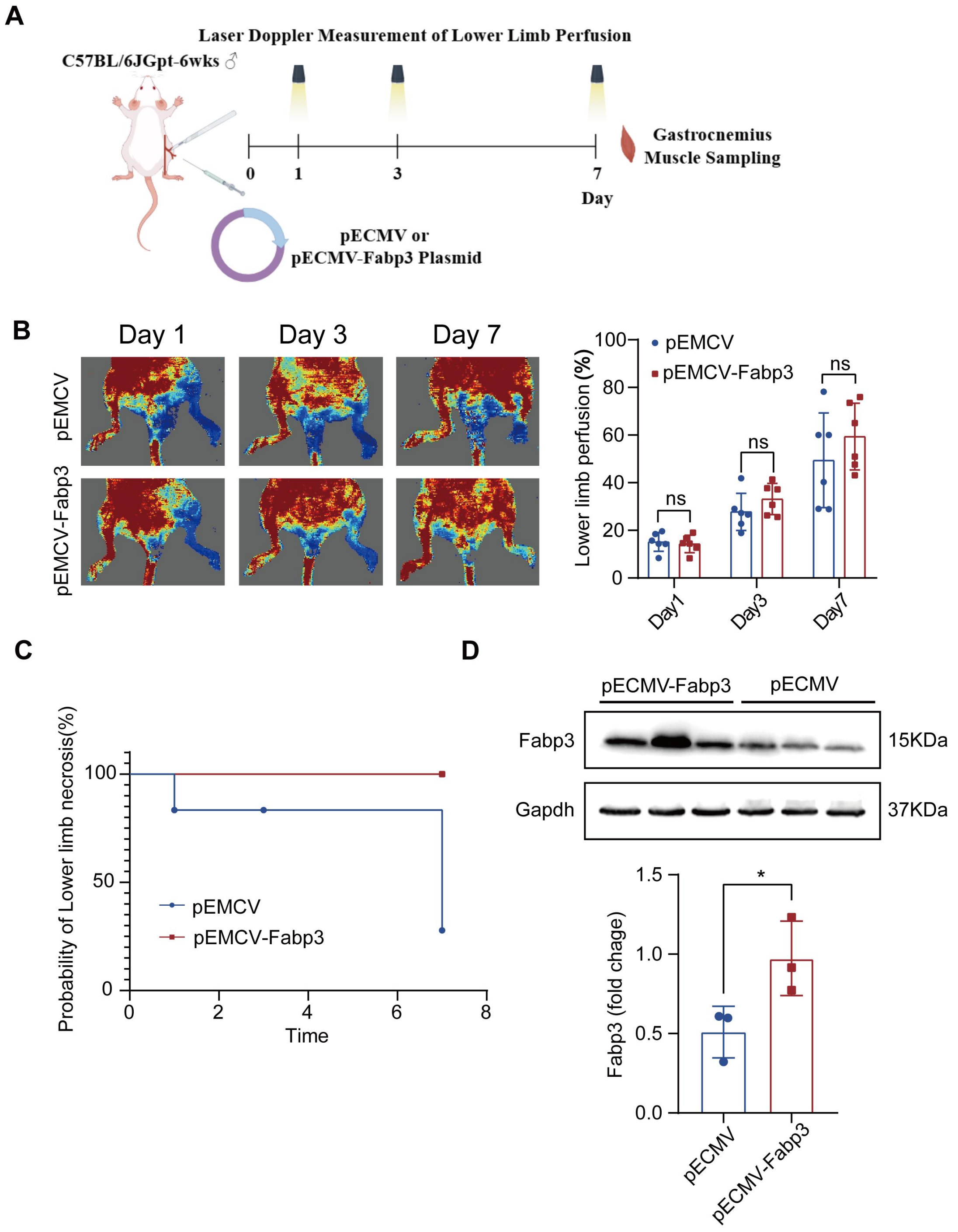

Fig.S4 Overexpression of FABP3 in mice with lower limb ischemia. A. The schematic diagram of the experimental procedures is presented. Ischemic lower limb muscles were infected with either pECMV or pECMV-Fabp3 plasmid. Blood perfusion was assessed on days 1, 3, and 7 post-infection, with samples collected on day 7. B. Blood perfusion images of the ischemic hindlimbs are shown for days 1, 3, and 7. Quantitative analysis of blood perfusion was conducted using two-way ANOVA, with n=6 in both the pECMV and pECMV-Fabp3 groups. C. The probability of lower limb necrosis is depicted. D. The expression levels of Fabp3 in the gastrocnemius muscle were analyzed by Western blotting across the two groups, with n=3 per group. *, $P < 0.05$.

Table S1. Clinical characteristics of Control group and AD without LLI

	Con (n=13)	AD without LLI (n=20)
Sex		
Male, n (%)	10 (76.9)	18(90.0)
Age	58±11.2	56±12

Table S2. Real-time PCR primer sequences

Name	Sequence (5'-3')	Species
Gapdh-F	AGGTCGGTGTGAACGGATTTG	Mouse
Gapdh-R	TGTAGACCATGTAGTTGAGGTCA	Mouse
Cast-F	GGAAGGACAAACCAGAGAAGC	Mouse
Cast-R	AGGGGCAGCTATCCAAATCTT	Mouse
Capn1-F	ATGACAGAGGAGTTAATCACCCC	Mouse
Capn1-R	GGCTATGAGAAACCGGAGGG	Mouse
Capn2-F	GGTCGCATGAGAGAGCCATC	Mouse
Capn2-R	CCCCGAGTTTTGCTGGAGTA	Mouse
Il-6-F	TAGTCCTTCCTACCCCAATTTCC	Mouse
Il-6-R	TTGGTCCTTAGCCACTCCTTC	Mouse
Il-10-F	GCTCTTACTGACTGGCATGAG	Mouse
Il-10-R	CGCAGCTCTAGGAGCATGTG	Mouse
Il-1β-F	GCAACTGTTCCTGAACTCAACT	Mouse
Il-1β-R	ATCTTTTGGGGTCCGTCAACT	Mouse
Tnf-α-F	GACGTGGAACTGGCAGAAGAG	Mouse
Tnf-α-R	TTGGTGGTTTGTGAGTGTGAG	Mouse
Mmp3-F	ACATGGAGACTTTGTCCCTTTTG	Mouse
Mmp3-R	TTGGCTGAGTGGTAGAGTCCC	Mouse
Mmp9-F	CTGGACAGCCAGACACTAAAG	Mouse
Mmp9-R	CTCGCGGCAAGTCTTCAGAG	Mouse
Tgf-β-F	CCACCTGCAAGACCATCGAC	Mouse
Tgf-β-R	CTGGCGAGCCTTAGTTTGGAC	Mouse
